# Evaluation of Quadruple Real-Time PCR Method to Detect Enterococci Carrying Vancomycin-Resistant Genes *vanA, vanB, vanM* in Rectal Swabs

**DOI:** 10.3389/fmed.2020.00403

**Published:** 2020-09-08

**Authors:** Yuanhui He, Genjie Ruan, Hui Hao, Feng Xue, Sainan Zhu, Bingbing Xiao, Bo Zheng

**Affiliations:** ^1^Institute of Clinical Pharmacology, Peking University First Hospital, Beijing, China; ^2^Department of Obstetrics and Gynecology, Peking University First Hospital, Beijing, China; ^3^IPE Biotechnology Co. Ltd, Beijing, China; ^4^Department of Biostatistics, Peking University First Hospital, Beijing, China

**Keywords:** quadruple real-time PCR method, rapid detection, enterococci carrying vancomycin resistant gene, genotyping, vancomycin heteroresistance enterococci carrying *vanM*, rectal swabs

## Abstract

**Purpose:** To evaluate the sensitivity and accuracy of the quadruple real-time PCR method for the detection of enterococci carrying vancomycin-resistant genes *vanA, vanB*, and *vanM* in rectal swabs.

**Methods:** Choosing PCR-sequenced DNA extracted directly from rectal swabs as the gold standard, the results of the quadruple real-time PCR method and the traditional method (screening culture combined PCR-sequencing method whose DNA extracted from single colony) were compared with the gold standard. The sensitivity, specificity, positive predictive value (PPV), negative predictive value (NPV), and accuracy of the quadruple real-time PCR method and the traditional method were obtained. The time required for the three methods was calculated.

**Results:** The results between gold standard and the quadruple real-time PCR method were similar. Compared to the traditional method, the quadruple real-time PCR method had a much higher sensitivity, specificity, PPV, NPV, and consistency. Our study found that the quadruple real-time PCR method is beneficial for detection of enterococci carrying *vanM* with vancomycin heteroresistance. The traditional method had high specificity and NPV, but its sensitivity and PPV were not ideal. The time needed for gold standard is a minimum of 28 h; the quadruple real-time PCR method takes 2–3 h while the traditional method consumes a minimum of 72 h.

**Conclusion:** The quadruple real-time PCR method can provide a rapid and reliable result for the diagnosis of patients with colonized vancomycin-resistant enterococci. This new method is beneficial for the active screening, timely clinical treatment measures, epidemiological research, and hospital monitoring of the enterococci carrying vancomycin-resistant gene, especially for the enterococci with vancomycin heteroresistance carrying *vanM*.

## Introduction

*Vancomycin-resistant enterococci* (VRE) infection is associated with increased treatment costs and prolonged hospitalization (1, 2). In China, the genotypes of VRE mainly consist of *vanA, vanB*, and *vanM*. Active screening, which mainly consists of the traditional culture method and the molecular detection method at present, can reduce the incidents of VRE infections. Recently, a new method was developed to simultaneously detect eight genes including *vanA, vanB*, and *vanM* genes in strain analysis (3). At the strain analytic stage, a highly accurate performance of the quadruple real-time PCR method was achieved (4). The reproducibility of the quadruple real-time PCR kit is ideal (variation coefficient <5%) and the limitations of detecting *vanA, vanB*, and *vanM* are all 100 CFU/mL (data not published). The objective of this study is to evaluate the performance of this novel multiple real time PCR assay in rectal swabs. To our knowledge, this is the first report to evaluate the quadruple real-time PCR method for enterococci carrying vancomycin-resistant genes *vanA, vanB*, and *vanM* simultaneously in clinical samples.

## Methods and Materials

### Patient Specimens

A total of 403 clinical rectal swabs transported by MW170 transwab (Medical Wire & Equipment, Corsham, Wales, England) from 403 different patients were submitted to the laboratory for genetic screening of vancomycin-resistant enterococci. They were tested to determine the clinical sensitivity, specificity, positive predictive value (PPV), and negative predictive value (NPV) of the quadruple real-time PCR method. Participants had to meet the following requirements to take part in this study: patient groups with a high risk of VRE colonization, especially for haemato-oncology and transplant patients and immune suppressed and critically ill patients in the ICU, by virtue of their intrinsic compromised immunity, antibiotic exposure, and their potential exposure to colonized patients (5, 6). The Institutional Review Board of Peking University First Hospital approved this study (2020 Research 202). Written informed consent from the participants' legal guardian/next of kin was not required to participate in this study in accordance with the national legislation and the institutional requirements.

### Control Group Setting

ATCC29212, BM4147, ZB212, and ZB17 were all *Enterococcus*. They are preserved by the Institute of Clinical Pharmacology of Peking University and their species and genotypes have been identified. BM4147, ZB212, and ZB17 carry *vanA, vanM*, and *vanB* genes, respectively. ATCC29212 carries none of the three genes. Every strain to be tested was recovered and purified before the experiment to ensure the viability and purity of the bacteria. Their DNA were extracted and used in the three methods as the control group. ATCC29212, BM4147, ZB212, ZB17, and the nuclease-free water were chosen as the negative control, *vanA* positive control, *vanM* positive control, *vanB* positive control, and blank control, respectively.

### Traditional Method

**Figure 1:**
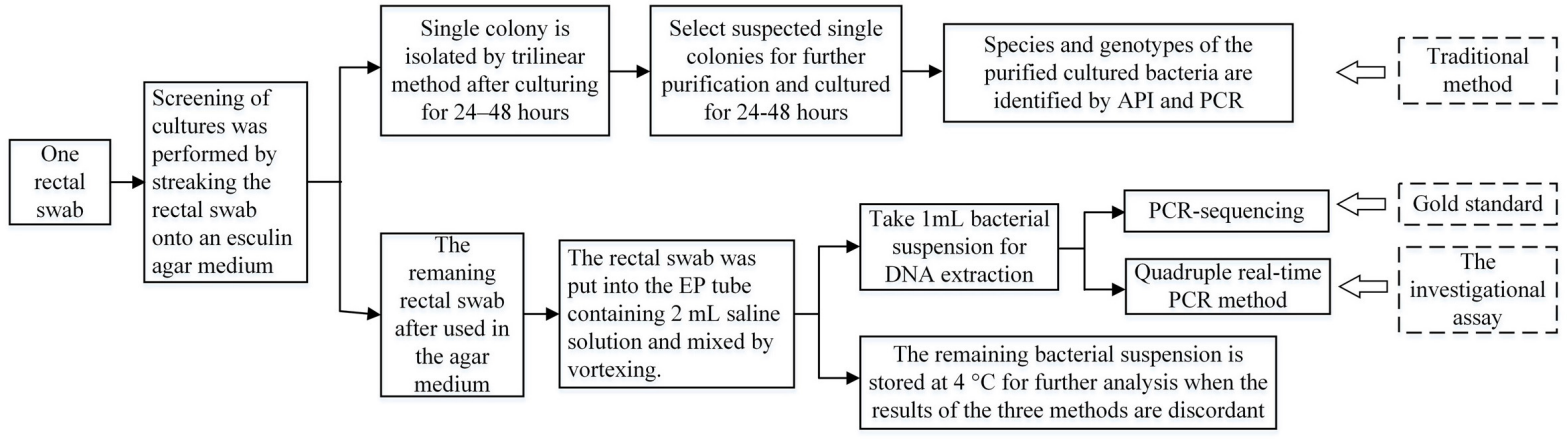
The procedure of the three methods in this study.

The screening culture was performed by streaking a rectal swab in trilinear method onto an esculin agar medium containing vancomycin (6 μg/mL) followed by incubation at 37°C for 24 or 48 h. Suspicious single colonies were transferred to blood plates containing vancomycin (6 μg/mL) for 24–48 h for purification culture, and was identified by API 32E tests. The extracted DNA was amplified by primers and then sequenced to identify their species and genotypes. After application of agar medium in the traditional method, the rectal swab was placed in an EP tube containing 2 mL saline solution and was mixed by vortexing. One milliliter bacterial suspension was taken for DNA extraction for both the gold standard and investigational assay, the remaining bacterial suspension was stored at 4°C for further analysis when the results of the three methods were discordant. The experimental procedure is shown in Figure 1.

### DNA Extraction

The DNA used in both the gold method and quadruple real-time PCR method was directly extracted from the rectal swabs using DNA extraction Kit (Bacterial Genome DNA Extraction Kit; TIANGEN, Catalog no. DP 302) according to the manufacturer's guidelines (Bacterial Genome DNA Extraction Handbook DP190814). Briefly, 1 mL of saline solution was taken from the EP tube containing an anal swab; 20 μL of internal control (2010806, IPE, Beijing, China) was added, mixed, and centrifuged at 10,000 rpm (~11,500 × g) for 1 min and the supernatant was discarded. Liquid amies buffer GA was added and mixed until homogenous suspension was achieved. The proteinase K and the liquid amies buffer GB were subsequently added to the suspension and incubated at 70°C for 10 min. Subsequently, 100% ethanol was added followed by centrifugation. DNA was then isolated following a series of centrifugations with TIANGEN spin columns and buffer solutions. DNA was eluted in 200 μL elution buffer (Buffer TE); quantity and quality was assessed using an enzyme-labeling measuring instrument. Extracted DNA was stored at −20°C for subsequent PCR and quadruple real-time PCR. The DNA extraction in the traditional method: A single colony was transferred into 200 μL saline solution and was boiled for 10 min at 100°C. After boiling, the liquid containing bacteria was centrifuged for 30 s with 12,000 r/s. Extracted DNA was stored at −20°C for subsequent PCR in the traditional method. The DNA extraction for control strains was the same as the traditional method.

### Gold Standard

The gold standard procedure consists of the DNA extraction and PCR-sequencing; the experimental procedure is shown in Figure 1. The reaction mixture (20 μL) consisted of 1 μL upstream primer (10 μmol/L), 1 μL downstream primer (10 μmol/L), 10 μL Taq PCR MasterMix (KT201, TIANGEN, Beijing, China), 6 μL sterile ultrapure water, and 2 μL DNA templates. The experimental procedures and primers of *vanA, vanB, vanM*, and van-N are previously described (3). Electrophoresis was carried out in 1.5% agarose gel at 110 V and 80 mA for 50 min; 200 bp DNA was used as a marker.

### The Investigational Assay

The investigational assay procedure consisted of the DNA extraction and the quadruple real-time PCR method as shown in Figure 1. The reaction mixture of quadruple real-time PCR consists of the primers and probes of *vanA, vanB, vanM*, and the internal control gene. The quadruple real-time PCR method was performed according to the manufacturer's instructions (2010806, IPE, Beijing, China). The DNA in the reaction was extracted directly from rectal swabs. The primers and experimental procedures were described previously (4). The results were analyzed according to the instructions of the manufacturer (quadruple real-time PCR kit).

### Discordant Result Analysis

Any specimen showing contradictory results by the traditional and gold standard methods would be re-cultured on the drug free medium and the species and genotypes would be identified by PCR sequencing. Any specimen that showed negative results with the quadruple real-time PCR method while positive in the gold standard would be tested again by the quadruple real-time PCR method and the result obtained was defined as the final result.

### Vancomycin Susceptibility

*Enterococcus* carrying vancomycin resistance genes was tested for vancomycin susceptibility by the agar dilution method and *E*-test (AB Biodisk, Solna, Sweden) to determine the source of the genes. The *Enterococcus* strains were identified as VRE with a vancomycin minimal inhibitory concentration (MIC) ≥32 μg/mL. The methods were all according to Clinical and Laboratory Standards Institute (CLSI) guidelines updated in 2018 (7).

### The Time Required for the Three Methods

The details of the time for the three methods: For the gold method and quadruple real-time PCR method: the starting point was set to the moment when DNA extraction from the rectal swab began, while the ending point were all set to the moment when the result was obtained. For the traditional method: the starting point was set to the moment when culture was started, while the ending point was set to the moment the result was obtained.

### Statistical Analysis

Fisher's exact test was used to calculate the sensitivity, specificity, PPV, NPV, and their 95% confidence intervals by GraphPad Prism 5.0 software (GraphPad Software, Inc., La Jolla, CA). The diagnostic accuracy and its 95% exact confidence intervals were calculated by Stata 9.0 (Stata Corp., College Station, TX, USA). The consistency was analyzed through the Kappa test method by SPSS 20.0 (IBM Corp., Armonk, NY, USA): the consistency is good if the Kappa coefficient is ≥0.75. The consistency is general if the Kappa coefficient is <0.75, ≥0.40. The consistency is not ideal if the Kappa coefficient is <0.40. *P* < 0.05(two-tailed) showing statistical significance.

## Results and Discussion

In this study, we developed a new method to detect the colonization of the enterococci carrying vancomycin-resistant gene. We tested 403 rectal swabs from 403 patients by the gold standard, the quadruple real-time PCR method and traditional method.

**Figure 2:**
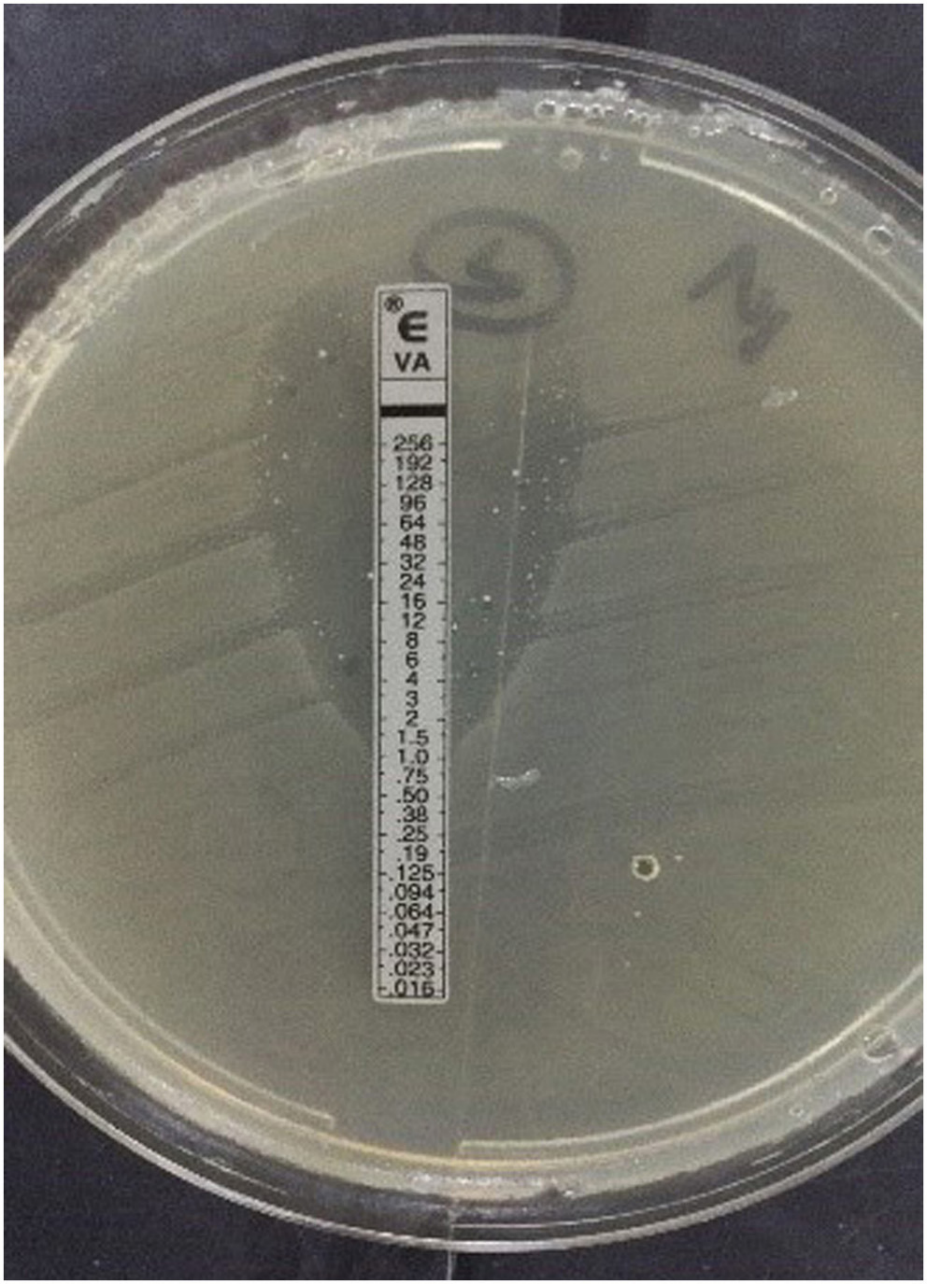
Vancomycin heterogeneous resistant *Enterococcus* carrying *vanM* obtained from antibiotic free medium.

Before the resolution of discordant results, a total of 19 rectal swabs showed differences among the three methods. These 19 rectal swabs were positive (carrying vancomycin resistance genes) by gold standard but were negative by the traditional method. One of the 19 rectal swabs (rectal swab 1-62) was *vanA* positive by the gold standard but was negative by the quadruple real-time PCR method. They were all tested again and analyzed for further resolution. Finally, two strains of vancomycin heterogeneous resistant *Enterococcus* carrying *vanM* (Figure 2) were obtained.

After resolution of discordant results, a total of 49 strains of *Enterococcus* containing vancomycin resistance genes were obtained by the traditional method. Forty-seven of those strains were VRE and two were vancomycin heterogeneous resistant *Enterococcus* carrying *vanM*. The VRE strains included 39 strains with *vanA* and 8 strains with *vanM*. These strains were obtained from the medium containing 6 μg /mL vancomycin, and their vancomycin MIC were all ≥32 μg/mL. The vancomycin heterogeneous resistant *Enterococcus* were obtained from the drug-free medium and their vancomycin MIC were 256 μg /mL by *E*-test.

**Figure 3:**
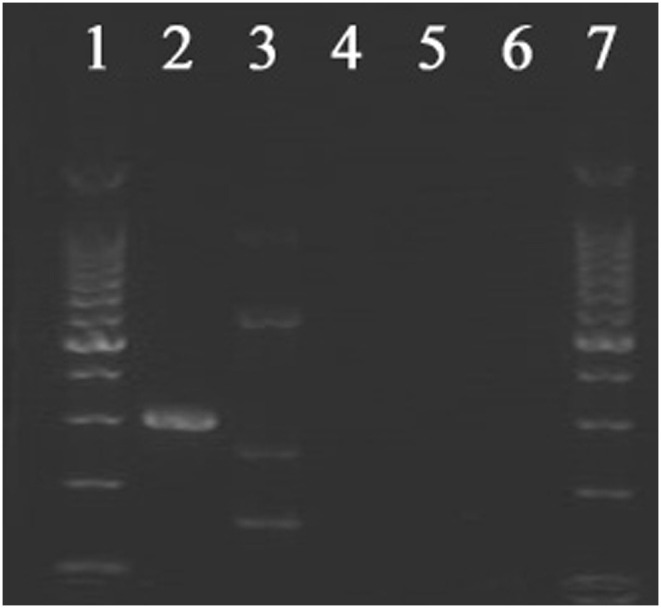
Electrophoresis results of DNA isolated from partial of rectal swabs amplified with *vanB* primers. Lanes 1 and 7 contain 200 bp Marker. Lanes 2, 3, and 4 represent positive control, negative control, and blank control of *vanB*, respectively. Lanes 5 and 6 show electrophoretic results of DNA of *Enterococcus* isolated from different rectal swabs amplified by *vanB* primers. Electrophoresis results showed that these strains do not carry *vanB*.

The results of the quadruple real-time PCR method were very similar to those of the gold standard. Except in one case, the rectal swab 1-62 was positive for *vanA* by the gold standard while negative by the quadruple real-time PCR method (this sample was also negative by the traditional method). None of the three methods detected the *Enterococcus* containing *vanB* gene. Partial electrophoresis results of the *vanB* gene are shown in Figure 3. More details about the number of samples containing vancomycin resistance genes are shown in Table 1.

**Table 1:** The results obtained by using three methods to detect *vanA, vanB*, and *vanM* in 403 c1linical rectal swabs after analysis of discordant results.

**Targeted gene**	**Gold standard**	**Multiple real-time PCR method**	**Traditional method**
Samples carrying *vanA* only	35	34	39
Samples carrying *vanB* only	0	0	0
Samples carrying *vanM* only	8	8	10^a^
Samples carrying *vanA* and *vanM* simultaneously	25	25	0
Total samples carrying vancomycin resistance genes	68	67	49
Total samples carrying no vancomycin resistance genes	335	336	354

a *10 strains include 8 strains of vanM VRE and 2 strains of vancomycin heterogeneous resistant Enterococcus carrying vanM*.

When we tested whether the samples only contained the *vanA* gene, 39 positive samples were obtained by the traditional method. While detection by the quadruple real-time PCR method and the gold standard revealed that 25 rectal swabs were positive, while the other 14 rectal swabs all contained both *vanA* and *vanM* genes, which were defined as negative.

When the samples were tested for the presence of the *vanM* gene alone, 10 positive samples were obtained by the traditional method. The same samples when detected by the quadruple real-time PCR method and the gold standard, obtained 4 positive and 6 negative samples, respectively. In the negative samples, 5 samples contained both *vanA* and *vanM*, while one sample (rectal swab 1-138) had no *vanM* detected by both the methods.

This may be due to the extremely limited amount of *vanM* present in that sample (1–138). After it was used in the traditional method, the amount of bacteria had reduced and could not reach the detection limit of the quadruple real-time PCR method and the gold standard.

When tested whether both *vanA* and *vanM* simultaneously existed in the same rectal swab, 25 samples were all positive by both the gold standard and the quadruple real-time PCR methods. The results obtained from the same samples by the traditional method revealed that 14 samples contained *vanA* only, 5 samples contained *vanM* only, and 6 samples didn't contain any vancomycin-resistant genes. A further resolution of discordant results showed that 6 rectal swabs did not contain the *Enterococcus* carrying vancomycin resistance genes after the re-culture and purification in the drug-free medium. However, one vancomycin heterogeneous resistant *Enterococcus* carrying *vanM* was isolated from rectal swab 2-024 by the same procedure. It is undeniable that the quadruple real-time PCR method has a higher sensitivity and specificity than the culture method. Only part of the samples' target strains could be isolated in the discordant results during resolution steps which might be because of inactivation of enterococci due to the long storage time of the rectal swabs.

Other reasons might be as follows: (i) Some *Enterococcus* strains containing both *vanA* and *vanM* were detected by the gold standard and quadruple real-time PCR method, but they could not be isolated by the traditional method. In the strain analysis step, the quadruple real-time PCR method can accurately detect 20 enterococci strains that contain both *vanA* and *vanM*, with a 100% accuracy (4). In this study, although, the *Enterococcus* containing both *vanA* and *vanM* were not isolated by the culture method, it is undeniable that rectal swabs may contain *Enterococcus* harboring both *vanA* and *vanM* when the results were *vanA* and *vanM* positive by the gold standard and quadruple real-time PCR methods.

(ii) *vanA* and *vanM* genes exist in different *Enterococcus* in the same rectal swab, respectively. However, the traditional method failed to isolate both genotypes of different *Enterococcus* in the same rectal swab. In subsequent studies, ZB366 (*vanM*) & ZB410 (*vanA*), ZB317 (*vanA*) & ZB407 (*vanM*), and ZB329 (*vanA*) & ZB36 (*vanM*) were isolated from anal swabs taken from three patients at two different time points in the week (data not included in the study). However, traditional methods have failed to isolate *Enterococcus* containing different vancomycin resistance genes in the same swab, but the result does not rule out that different *Enterococcus* containing *vanA* and *vanM* genes separately may exist in the same patient at the same time. The reasons for the different vancomycin-resistant *Enterococcus* genes isolated from the rectal swabs collected at different time points from the same patient are still to be fully explored, which may be due to human factors having a greater impact on the results obtained by traditional methods compared to the other two methods or the different proportion of *Enterococcus* with different vancomycin resistance genes at different time points.

(iii) *vanA* or *vanM* genes were found in other strains but not from *Enterococcus* in some samples. However, this is less likely as the vancomycin resistance genes were not detected in 90 non-enterococci strains in the strain analysis step (4).

Compared to the traditional method, the quadruple real-time PCR method is confirmed to have a much higher sensitivity, specificity, PPV, NPV, and accuracy. When the *vanA, vanB*, and *vanM* genes were detected separately, the kappa coefficients between the gold standard and quadruple real-time PCR method were all nearly equal to 1. The traditional method has high specificity and NPV, but its sensitivity and PPV are not so ideal; more details are shown in Table 2.

**Table 2:** Comparison of quadruple real-time PCR and traditional methods to detect *vanA, vanB, vanM* in 403 clinical rectal swabs.

		**No. of isolates with each set of results**									
		**Reference** **+**		**Reference –**							
		**Test +**	**Test —**	**Test +**	**Test –**	**Sensitivity**	**Specificity**	**PPV**	**NPV**	**Accuracy**	**Kappa coefficient(*P*)**
Multiple real-time PCR method	*vanA*	34	1	0	368	97.14% (85.47, 99.85^a^)	100% (98.97, 100^a^)	100% (89.85, 100^a^)	99.75% (98.63, 99.99^a^)	99.76% (98.66, 99.99^a^)	0.984 (<0.001)^*^
	*vanM*	8	0	0	395	100% (67.56, 100.0^a^)	100% (99.04, 100.0^a^)	100% (67.56, 100.0^a^)	100% (99.09, 100.0^a^)	100.00% (99.11, 100.00^a^)	1 (<0.001)^*^
	*vanA*+ *vanM*	25	0	0	378	100% (86.68, 100.0^a^)	100% (98.99, 100.0^a^)	100% (86.68, 100.0^a^)	100% (99.09, 100.0^a^)	100.00% (99.11, 100.00^a^)	1 (<0.001)^*^
Traditional method	*vanA*	25	10	14	354	71.43% (54.95, 83.67^a^)	96.2% (93.72, 97.72^a^)	64.1% (48.42, 77.26^a^)	94.04% (91.27, 96.15^a^)	94.17% (91.46, 96.23^a^)	0.643 (<0.001)^*^
	*vanM*	4	4	6	389	50% (21.52, 78.48^a^)	98.48% (96.73, 99.3^a^)	40.0% (16.82, 68.73^a^)	97.51% (95.48, 98.8^a^)	97.57% (95.58, 98.8 ^a^)	0.432 (<0.001)^*^
	*vanA*+ *vanM*	0	25	0	378	0.0% (0.0, 13.32^a^)	100% (98.99, 100^a^)	–(0, 1^a^)	93.79% (90.98, 95.95^a^)	93.93% (91.17, 96.04^a^)	0.0(1.00)

*reference, gold standard. reference +, positive result for the reference. test +, positive result for the indicated test*.

a *95% confidence interval*.

* *the consistency is good. PPV, positive predictive value. NPV, negative predictive value. vanA, the sample carries vanA only. vanB, the sample carries vanB only. vanM, the sample carries vanM only. vanA+ vanM, the sample carries vanA and vanM gene simultaneously*.

The time needed for the gold standard (PCR-sequencing whose DNA was directly extracted from the swabs) is 28 h minimum, while the quadruple real-time PCR method takes ~2–3 h; the traditional method (culture combined with PCR-sequencing) takes 72 h at least.

For the three methods, the average extraction time of DNA from a sample was 30 min. The gold standard method includes DNA extraction, PCR, DNA cleavage agarose gel electrophoresis, and sequencing for the last identification. The quadruple real-time PCR method includes DNA extraction and quadruple real-time PCR. The time cost of PCR-sequencing and quadruple real-time PCR were as described previously [3]. The traditional method includes screening the purification of the culture (24 h at least), DNA extraction, and identification of genotype and species by PCR-sequencing.

In some studies, associated with the evaluation of molecular methods to detect VRE in clinical samples, *vanB* is the most common in their positive results. However, the amount decreased significantly after culture reconfirmation (8–10). The reasons for this are speculated as follows: (i) The *vanB* gene may exist in non-*Enterococcus* (8, 11). (ii) The specificity of some methods needs to improve. The *VanB* gene was not detected by any of the three methods probably due to the time and place of swab collection being relatively limited. Although the evaluation of the quadruple real-time PCR method on *vanB* was not obtained in the clinical analysis stage, it can accurately distinguish the *vanB* gene from other genotypes in the strain analysis stage (4). It demonstrated that our quadruple real-time PCR method may have a high specificity in *vanB* gene detection in clinical samples.

Unlike *vanM* VRE with high resistance to vancomycin which appeared in previous studies (12–14), enterococci carrying *vanM* with vancomycin heteroresistance has rarely been reported all over the world. The vancomycin heteroresistance enterococci carrying *vanM* is important due to its great clinical value. Conventional antibiotic susceptibility tests may miss detecting them, and subsequently mistake vancomycin sensitive strains leading to vancomycin treatment failures impairing hospital infection prevention and control. This further illustrates the importance of the detection of *vanM* gene by this quadruple real-time PCR method.

The advantages of the quadruple real-time PCR method are as follows: (i) Compared to the tedious traditional culture method, this new method provides results in only ~2–3 h. (ii) avoids the misdiagnosis of vancomycin heteroresistance enterococci carrying *vanM* by traditional culture (15).

Our study has several advantages as follows: (i) Screening culture medium containing vancomycin 6 μg/mL, which is more sensitive compared to one medium with 32 μg/mL vancomycin (16). (ii) Although the swab causes discomfort to patients, it can be obtained immediately. (iii) The usage of rectal swabs can reduce the repetition of experiments compared to fecal samples, as some substances in fecal matter can inhibit the quadruple real-time PCR reaction (10). (iv) The traditional method for screening VRE has a more accurate result when cultured in 48 h than in 24 h (16). (v) The adoption of internal control for screening the extraction of DNA and the whole process of PCR amplification, which is necessary for the judgement of the false negative results caused by the action of the inhibitor (17, 18). (vi) The evaluation of these new methods is more objective as we chose two different detection methods as the reference methods. (vii) We re-cultured the clinical samples on the antibiotic-free medium to further analyze inconsistent results. This improves the accuracy of the traditional method.

However, as the rectal swabs had been stored for a long time when we re-cultured on the drug-free medium, some *vanM* vancomycin heterogeneous resistant enterococci may have been inactivated, reducing the accuracy of the traditional method.

## Conclusion

In summary, the quadruple real-time PCR method is ideal for obtaining the results quickly and differentiating *vanA, vanB*, and *vanM accurately* in rectal swabs. This method is of great clinical significance for the detection of vancomycin heteroresistance *Enterococcus* carrying *vanM*, and it is beneficial for active screening, taking timely clinical treatment measures, epidemiological research, and hospital monitoring of enterococci carrying vancomycin-resistant genes (19).

## Data Availability Statement

All datasets generated for this study are included in the article/supplementary material.

## Ethics Statement

The Institutional Review Board of Peking University First Hospital approved this study (2020 Research 202). Written informed consent from the participants' legal guardian/next of kin was not required to participate in this study in accordance with the national legislation and the institutional requirements.

## Author Contributions

YH, GR, HH, BX, and BZ conceived the study design. YH and GR were responsible for the recruitment and collection of samples. YH, GR, HH, and FX were responsible for the laboratory assays. YH and SZ performed the data analysis. YH completed the initial manuscript. YH, GR, and BZ revised the manuscript. All authors read and approved the final version of the manuscript.

## Conflict of Interest

HH was employed by the company IPE Biotechnology Co. Ltd, Beijing, China. The remaining authors declare that the research was conducted in the absence of any commercial or financial relationships that could be construed as a potential conflict of interest.

## References

[1] Cheng VC Chen JH Tai JW Wong SC Poon RW Hung IF. Decolonization of gastrointestinal carriage of vancomycin-resistant *Enterococcus* faecium: case series and review of literature. BMC Infect Dis. (2014) 14:514. 10.1186/1471-2334-14-514 25248287 PMC4180964

[2] Mutters NT Mersch-Sundermann V Mutters R Brandt C Schneider-Brachert W Frank U. Control of the spread of vancomycin-resistant Enterococci in hospitals. Deutsch Aerztebl Int. (2013) 110:725–31. 10.3238/arztebl.2013.0725 24222791 PMC3822708

[3] Nomura T Hashimoto Y Kurushima J Hirakawa H Tanimoto K Zheng B. New colony multiplex PCR assays for the detection and discrimination of vancomycin-resistant Enterococcal species. J Microbiol Methods. (2018) 145:69–72. 10.1016/j.mimet.2017.12.013 29309802

[4] He YH Ruan GJ Hao H Xue F Ma YK Zhu SN. Real-time PCR for the rapid detection of vanA, vanB and vanM genes. J Microbiol Immunol Infect. (2019) 10.1016/j.jmii.2019.02.002. [Epub ahead of print]. 30926279

[5] Humphreys H. Controlling the spread of vancomycin-resistant Enterococci. is active screening worthwhile? J Hosp Infect. (2014) 88:191–8. 10.1016/j.jhin.2014.09.002 25310998

[6] Isenman H Fisher D. Advances in prevention and treatment of vancomycin-resistant *Enterococcus* infection. Curr Opin Infect Dis. (2016) 29:577–82. 10.1097/QCO.0000000000000311 27584589

[7] Clinical and Laboratory Standards Institute Performance Standards for Antimicrobial Susceptibility Testing: Twentieth Informational Supplement M100-S20. Wayne, PA: CLSI (2018).

[8] Babady NE Gilhuley K Cianciminio-Bordelon D Tang YW. Performance characteristics of the Cepheid Xpert vanA assay for rapid identification of patients at high risk for carriage of vancomycin-resistant Enterococci. J Clin Microbiol. (2012) 50:3659–63. 10.1128/JCM.01776-12 22972822 PMC3486258

[9] Zhou X Arends JP Kampinga GA Ahmad HM Dijkhuizen B van Barneveld P. Evaluation of the Xpert vanA/vanB assay using enriched inoculated broths for direct detection of vanB vancomycin-resistant Enterococci. J Clin Microbiol. (2014) 52:4293–7. 10.1128/JCM.01125-14 25297325 PMC4313300

[10] Bae MH Kim J Sung H Jeong YS Kim MN. Evaluation of iNtRON VRE vanA/vanB real-time PCR for follow-up surveillance of VRE-infected or colonized patients. Diagn Microbiol Infect Dis. (2013) 77:292–5. 10.1016/j.diagmicrobio.2013.08.006 24094836

[11] Graham M Ballard SA Grabsch EA Johnson PD Grayson ML. High rates of fecal carriage of nonenterococcal vanB in both children and adults. Antimicrob Agents Chemother. (2008) 52:1195–7. 10.1128/AAC.00531-07 18180361 PMC2258543

[12] Xu X Lin D Yan G Ye X Wu S Guo Y. vanM, a new glycopeptide resistance gene cluster found in *Enterococcus* faecium. Antimicrob Agents Chemother. (2010) 54:4643–7. 10.1128/AAC.01710-09 20733041 PMC2976141

[13] Teo JW Krishnan P Jureen R Lin RT. Detection of an unusual van genotype in a vancomycin-resistant *Enterococcus* faecium hospital isolate. J Clin Microbiol. (2011) 49:4297–8. 10.1128/JCM.05524-11 21998432 PMC3232975

[14] Chen C Sun J Guo Y Lin D Guo Q Hu F. High Prevalence of vanM in Vancomycin-Resistant *Enterococcus* faecium Isolates from Shanghai, China. Antimicrob Agents Chemother. (2015) 59:7795–8. 10.1128/AAC.01732-15 26369966 PMC4649207

[15] Thaker MN Kalan L Waglechner N Eshaghi A Patel SN Poutanen S. Vancomycin-variable Enterococci can give rise to constitutive resistance during antibiotic therapy. Antimicrob Agents Chemother. (2015) 59:1405–10. 10.1128/AAC.04490-14 25512425 PMC4325790

[16] Wijesuriya TM Perry P Pryce T Boehm J Kay I Flexman J. Low vancomycin MICs and fecal densities reduce the sensitivity of screening methods for vancomycin resistance in Enterococci. J Clin Microbiol. (2014) 52:2829–33. 10.1128/JCM.00021-14 24871216 PMC4136124

[17] Wang X Liu F Jiang L Bao Y Xiao Y Wang H. Use of chimeric influenza viruses as a novel internal control for diagnostic rRT-PCR assays. Appl Microbiol Biotechnol. (2016) 100:1667–76. 10.1007/s00253-015-7042-y 26474983 PMC7080162

[18] Janse I Hamidjaja RA Hendriks AC van Rotterdam BJ. Multiplex qPCR for reliable detection and differentiation of Burkholderia mallei and Burkholderia pseudomallei. BMC Infect Dis. (2013) 13:86. 10.1186/1471-2334-13-86 23409683 PMC3579680

[19] Tan TY Jiang B Ng LS. Faster and economical screening for vancomycin-resistant Enterococci by sequential use of chromogenic agar and real-time polymerase chain reaction. J Microbiol Immunol Infect. (2015) 50:448–53. 10.1016/j.jmii.2015.08.003 26442675

